# Pregnancy Following Clam Cystoplasty and Mitrofanoff Procedure in a Patient With Fowler’s Syndrome and Detrusor Overactivity

**DOI:** 10.7759/cureus.89423

**Published:** 2025-08-05

**Authors:** Humaira S Malik, Salma Saad

**Affiliations:** 1 Department of Obstetrics and Gynecology, Dumfries and Galloway Royal Infirmary, Dumfries, GBR

**Keywords:** clam cystoplasty, fowler's syndrome, intermittent self-cathetrization, mitrofanoff, pregnancy

## Abstract

Fowler’s syndrome causes urinary retention due to failure of the urethral sphincter to relax. Management aims for complete bladder emptying, typically via intermittent self-catheterization. If conservative treatment fails, detrusor overactivity with Fowler’s syndrome may be managed surgically using clam cystoplasty and the Mitrofanoff procedure to increase bladder capacity and reduce pressure. We report a rare case of pregnancy in a woman with Fowler’s syndrome and detrusor overactivity, who previously underwent clam cystoplasty and Mitrofanoff procedure. She had a continent, functional abdominal stoma for clean intermittent catheterization. Her pregnancy was complicated by recurrent urinary tract infections and bladder spasms requiring antibiotics. A planned cesarean section was performed at 35 weeks following steroid administration and discussion with the multidisciplinary team, resulting in the delivery of a live baby. This case illustrates pregnancy management complexities in these women, focusing on the need for individualized, multidisciplinary care.

## Introduction

Fowler's syndrome is a rare urological condition characterized by difficulty urinating due to the abnormal function of the urethral sphincter, leading to chronic urinary retention [1]. It predominantly affects young women, particularly those with a history of trauma, polycystic ovaries, or gynecological or pelvic floor surgery. Neurological and urological conditions are excluded to necessitate the diagnosis [1]. The primary concern in managing this condition is ensuring regular bladder emptying, which is achieved through intermittent catheterization [2]. Fowler syndrome is typically characterized by detrusor underactivity due to poor relaxation of the urethral sphincter [3]. Detrusor overactivity may occasionally coexist. This adds further complexity to the clinical picture, posing significant quality-of-life issues and often necessitating multimodal management.

When a bladder is overactive or has poor compliance and does not respond to conservative treatments, such as anticholinergic drugs or intravesical botulinum toxin injections, augmentation cystoplasty may be considered as a next step [4]. Clam cystoplasty is a type of bladder augmentation surgery in which the bladder is opened and a segment of bowel is incorporated to enlarge the bladder capacity [5]. Patients who require this procedure often experience difficulties with bladder emptying or bladder outlet obstruction and may benefit from the creation of a catheterizable channel in addition to bladder augmentation [4]. The Mitrofanoff procedure involves creating a conduit (from the appendix or a segment of the small intestine) between the bladder and the skin, allowing for intermittent self-catheterization to manage urinary retention.

In this case report, we present a rare instance of pregnancy in a woman with Fowler’s syndrome and detrusor overactivity, managed surgically with clam cystoplasty and a Mitrofanoff channel.

## Case presentation

A 39-year-old woman, with no live births and previous two pregnancies resulting in miscarriage, attended the antenatal clinic at eight weeks of gestation following a spontaneous conception. She had a history of complex urological surgeries. She had a history of Fowler’s syndrome, diagnosed in her early 20s, and an overactive bladder. She had previously undergone placement of a sacral nerve stimulator 14 years ago, which was removed due to trauma-related malfunction. She was unsuccessfully treated with anticholinergics. She subsequently underwent clam cystoplasty and a right-sided Mitrofanoff procedure using a bowel segment 10 years ago, followed by revision surgery one year later. She had also received intravesical botulinum toxin injection in the bladder every six months.

She had a well-functioning urinary channel that permitted intermittent bladder drainage using aseptic catheterization via an abdominal stoma situated in the right iliac fossa. She has experienced over 85 hospital admissions and has a history of multiple antibiotic allergies, including allergies to cefexin, ciprofloxacin, penicillin, trimethoprim, and teicoplanin. She also has posttraumatic stress disorder related to healthcare experiences and trust issues with medical providers. Additionally, she has pernicious anemia and receives monthly vitamin B12 injections.

She has gestational diabetes, managed with insulin, and uses a blood glucose sensor. Her BMI is 40. She reports an increased need for catheterization due to the effects of pregnancy. She uses a hydrophilic-coated intermittent catheter, size 14, with an attached drainage bag. She experienced two episodes of vaginal bleeding during this pregnancy, at six and eleven weeks, for which she received progesterone treatment. Ultrasound imaging was challenging due to scar tissue; however, her routine detailed anomaly scan was normal, and serial growth scans showed normal fetal development.

Her care was individualized and was managed by a multidisciplinary team (MDT) comprising an obstetrician, diabetologist, microbiologist, and urologist. She was regularly screened for hypertension, and urine cultures were performed to detect and treat urinary tract infection (UTI) promptly. Her renal function was closely monitored for any signs of deterioration. Prophylactic antibiotics were administered during pregnancy to help prevent UTIs. Her renal function was normal.

She had multiple admissions during pregnancy under the obstetric team due to recurrent UTI and experienced significant pain and bladder spasms. She also found it increasingly difficult to pass a catheter. UTIs were managed with antibiotics guided by sensitivities and her allergy profile. Treatment options were limited due to a severe penicillin allergy and additional antibiotic allergies. Despite these challenges, she continued self-catheterization through the Mitrofanoff stoma.

**Figure 1:**
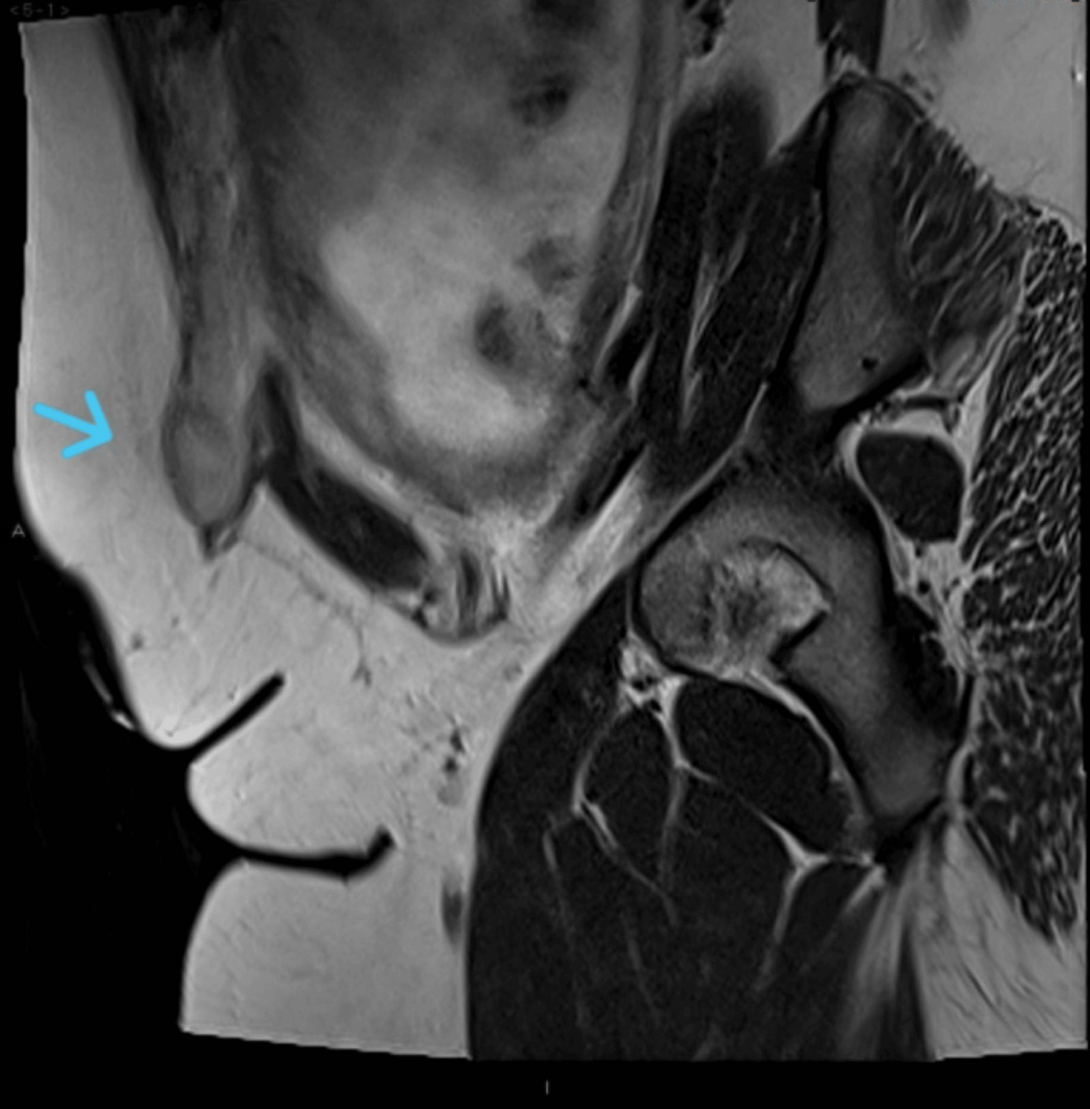
Right lower quadrant stoma in keeping with the known Mitrofanoff procedure (arrow)

A prophylactic dose of low-molecular-weight heparin (LMWH), specifically enoxaparin 60 mg subcutaneously once daily, was initiated at 28 weeks of gestation due to an intermediate risk of venous thromboembolism, with plans to continue it for six weeks postpartum. She was concerned about preserving the function of her Mitrofanoff stoma and understandably wished to avoid any damage or loss of function. She requested a planned cesarean section (CS) due to the unpredictability of labor onset and the need for appropriate staff, including a urologist, to be available in case of an emergency. The patient preferred an early scheduled caesarean delivery. An MRI of the pelvis and abdomen was performed to assess the anatomy and any structures overlying the uterus to aid in surgical planning, as shown in Figure 1.

Following an MDT discussion, delivery was planned at 35 weeks. She was admitted two days before the CS and received two doses of betamethasone 12 mg intramuscularly every 24 hours for fetal lung maturity. A planned CS was performed by a consultant obstetrician, with assistance from a urologist. The CS was performed via midline abdominal incision. Adhesions were noted, likely due to previous surgical interventions. There were no intraoperative complications.

A healthy neonate was delivered (APGAR 8/9) without complications. The Mitrofanoff stoma remained intact and functional. On postpartum day 1, she was diagnosed with bilateral pulmonary emboli and commenced on therapeutic LMWH for three months. She was discharged on postoperative day 4. On day 5, she presented with serous wound discharge approximately 3 cm from the lower edge of the midline incision. Initially managed conservatively as there was no evidence of infection, she later underwent incision and drainage under local anesthesia, during which serous fluid and a small amount of pus were drained. The wound subsequently healed well.

## Discussion

This case report discusses the challenges of pregnancy and delivery in a woman with Fowler’s syndrome and an overactive bladder, who has undergone complex urological reconstructive surgeries. When both bladder storage and voiding functions are impaired, combining augmentation cystoplasty with the creation of a continent catheterizable channel may be considered, provided the patient is appropriate [6]. However, for carefully selected patients, these reconstructive options can offer enhanced bladder holding capacity, better bladder control, protection of kidney function, and an overall improvement in quality of life [6].

Pregnancy after urinary tract reconstruction poses challenges, including preventing UTI, maintaining self-catheterization, and avoiding damage to urological modifications during delivery. A systematic review reported that the complication most commonly observed during pregnancy was febrile UTI [7,8]. Performing intermittent catheterization to empty the continent urinary diversion may cause bacteria to grow in the lower urinary tract and persistent bacteriuria [9]. A regular bacteriological testing of catheterized urine samples is recommended to detect infections early and allow prompt antibiotic treatment, helping to reduce the risk of pyelonephritis and preterm labor [10]. In this population, antibiotic prophylaxis during pregnancy is recommended. Research on weekly oral cyclic antibiotic prophylaxis has demonstrated that preventing asymptomatic bacteriuria in women using intermittent self-catheterization can effectively reduce the incidence of symptomatic or febrile UTI and lower the risk of low birth weight [11]. Another study also reported pregnancy in women after augmentation cystoplasty complicated by UTIs, and antibiotic prophylaxis was used thereafter during the rest of the pregnancy [10]. Some studies reported transient self-catheterization difficulties during pregnancy [5,7]. Literature review shows these difficulties were more common when the stoma was located in the right iliac fossa rather than at the umbilical site [7,12].

A review of the literature indicates no consensus on a particular mode of delivery as the best for women with urinary diversion or augmentation cystoplasties [13]. Careful planning of the mode of delivery is essential in women with lower urinary tract reconstruction. In this case report, the patient requests planned CS as she wants to preserve the continent stoma and avoid impairing the continence mechanism because of the unpredictability of the onset of labor and the availability of appropriate staff in the event of an emergency CS. As noted by Huck et al., a planned CS may be preferred due to concerns about the potential effects of vaginal delivery on urinary tract reconstruction [14]. Emergency CS can be more challenging due to intraperitoneal scarring and carries a higher risk of injury to prior urological reconstruction [15]. A higher CS rate was reported in other studies in patients with augmentation cystoplasty [11] and continent diversions [8]. In contrast, a successful vaginal delivery in a woman with a clam ileocystoplasty and appendix Mitrofanoff procedure was also reported [16].

Whenever possible, in all such cases with reconstructive surgery of the lower urinary tract, the presence of an experienced urologist is recommended to prevent damage to the augmentation cystoplasty and/or the continent catheterizable channel, enabling the obstetrician to direct attention to the delivery of the baby [8]. Earlier, concerns have been expressed relating to direct injury to the neobladder or its blood supply and difficulties or delays in reaching the uterus [17,18]. It is advisable to insert a catheter into a continent stoma during CS to prevent its injury [8]. The choice of surgical access for cesarean delivery may help minimize surgical complications. A midline abdominal incision is advised following bladder augmentation and Mitrofanoff stoma to minimize the risk of injury to the augmented bladder. This approach also provides better exposure for identification of the Mitrofanoff channel, ensuring it was not inadvertently transected during the procedure [7,18]. This case demonstrates that successful pregnancy outcomes are achievable through proactive MDT planning. Other studies support this finding [6-8,19].

Limited literature is available on pregnancy in Fowler's syndrome and Detrusor overactivity with post-Mitrofanoff and clam cystoplasty. Our literature search suggests that this is the first reported case of its kind to be published. We hope it contributes to the expanding clinical knowledge supporting successful pregnancy outcomes in this complex patient population.

## Conclusions

Successful pregnancy outcomes are achievable in women with Fowler’s syndrome and complex urological reconstruction; however, these cases present significant medical and surgical challenges. Individualized, multidisciplinary care is essential, particularly given the altered pelvic anatomy from procedures such as clam cystoplasty and Mitrofanoff formation. Planned delivery with urological input is pivotal to preserving prior surgical interventions. In cases of anatomical distortion due to prior surgeries, MRI offers valuable guidance for surgical planning. Recurrent UTIs and restricted antibiotic options during pregnancy further complicate management, emphasizing the need for early involvement of microbiology teams. These findings highlight that, while favorable outcomes are possible, they require meticulous planning, experienced teams, and acknowledgment of the considerable risks and technical complexity involved.

## References

[1] Rodrigues RF Sr, Antonini M, Toledo ML, Silva EK, Silva HF (2025). Case report of management of Fowler's syndrome during pregnancy: challenges and outcomes. Cureus.

[2] Khan AT, Kinder RB, Hayman RG (2010). Fowler's syndrome and pregnancy. BMJ Case Rep.

[3] Szymański JK, Słabuszewska-Jóźwiak A, Jakiel G (2021). Fowler's syndrome-the cause of urinary retention in young women, often forgotten, but significant and challenging to treat. Int J Environ Res Public Health.

[4] Stewart JN, Boone TB (2013). The contemporary indications for augmentation cystoplasty. Curr Bladder Dysfunct Rep.

[5] Cheng PJ, Myers JB (2020). Augmentation cystoplasty in the patient with neurogenic bladder. World J Urol.

[6] Holm HV, Martins FE, McCammon KA, Sandhu JS (2023). Bladder augmentation and urinary diversion. Female Genitourinary and Pelvic Floor Reconstruction.

[7] Bey E, Perrouin-Verbe B, Reiss B, Lefort M, Le Normand L, Perrouin-Verbe MA (2021). Outcomes of pregnancy and delivery in women with continent lower urinary tract reconstruction: systematic review of the literature. Int Urogynecol J.

[8] Biers SM, Venn SN, Greenwell TJ (2012). The past, present and future of augmentation cystoplasty. BJU Int.

[9] Natarajan V, Singh G (2000). Urinary diversion for incontinence in women. Int Urogynecol J Pelvic Floor Dysfunct.

[10] Salomon J, Schnitzler A, Ville Y, Laffont I, Perronne C, Denys P, Bernard L (2009). Prevention of urinary tract infection in six spinal cord-injured pregnant women who gave birth to seven children under a weekly oral cyclic antibiotic program. Int J Infect Dis.

[11] Greenwell TJ, Venn SN, Creighton S, Leaver RB, Woodhouse CR (2003). Pregnancy after lower urinary tract reconstruction for congenital abnormalities. BJU Int.

[12] Fenn N, Barrington JW, Stephenson TP (1995). Clam enterocystoplasty and pregnancy. Br J Urol.

[13] Huck N, Schweizerhof S, Stein R, Honeck P (2020). Pregnancy following urinary tract reconstruction using bowel segments: a review of published literature. World J Urol.

[14] Azanu WK, Sakyi AT, Adamu AF, Obeng F (2024). Successful pregnancy and delivery in a patient with a Mainz-II pouch urinary diversion: case report and literature review. Case Rep Womens Health.

[15] Bey E, Manach Q, Peyronnet B (2020). Pregnancy and delivery in women with lower urinary tract reconstruction: a national multicenter retrospective study from the French-Speaking Neuro-Urology Study Group (GENULF) and the Neuro-Urology Committee of the French Association of Urology. J Urol.

[16] Muthulakshmi B, Allahdin S (2010). Pregnancy with clam ileocystoplasty and appendix Mitrofanoff procedure: vaginal delivery can be considered. Int Urogynecol J.

[17] Correia C, Pardal C, Igreja J (2015). Management of pregnancy after augmentation cystoplasty. BMJ Case Rep.

[18] Güzel AI, Tokmak A, Kara AS, Yalinkaya A (2014). Spontaneous pregnancy after cystectomy and urinary diversion by Indiana pouch method for congenital bladder exstrophy: a case report. Br J Med Med Res.

[19] Ebert AK, Falkert A, Hofstädter A, Reutter H, Rösch WH (2011). Pregnancy management in women within the bladder-exstrophy-epispadias complex (BEEC) after continent urinary diversion. Arch Gynecol Obstet.

